# Metal-Catalyzed Carbon Foams Synthesized from Glucose as Highly Efficient Electromagnetic Absorbers

**DOI:** 10.3390/ma17143488

**Published:** 2024-07-14

**Authors:** Guan-Hong Liu, Chuan-Ying Wei, Ting Huang, Fei Wang, Jiang-Fan Chang, Qian Sun, Xian-Hui Zhang

**Affiliations:** 1 School of Marine Engineering, Jimei University, Xiamen 361021, Chinajf.chang@jmu.edu.cn (J.-F.C.); zhangxianhui@jmu.edu.cn (X.-H.Z.); 2 Xiamen Branch of the Luoyang Ship Material Research Institute, Xiamen 361021, China; 3 Xiamen Key Laboratory of Marine Corrosion and Smart Protective Materials, Fujian Provincial Key Laboratory of Naval Architecture and Ocean Engineering, Jimei University, Xiamen 361021, China

**Keywords:** glucose carbon foam, electromagnetic absorbing materials, metal catalysis

## Abstract

This paper introduces a novel method for preparing high-performance, metal-containing carbon foam wave-absorbing materials. The process involves foaming glucose through catalysis by transition metals followed by high-temperature pyrolysis. The resulting carbon foam materials exhibit a highly porous structure, which is essential for their wave-absorption properties. Notably, at a thickness of 2.0 mm, the glucose-derived carbon foam composite catalyzed by Fe and Co (GCF-CoFe) achieved a minimum reflection loss (*RL*_min_) of −51.4 dB at 15.11 GHz, along with an effective absorption bandwidth (EAB) of 5.20 GHz, spanning from 12.80 GHz to 18.00 GHz. These impressive performance metrics indicate that this approach offers a promising pathway for developing low-density, efficient carbon foam materials for wave-absorption applications. This advancement has significant implications for fields requiring effective electromagnetic interference (EMI) shielding, stealth technology, and other related applications, potentially leading to more efficient and lightweight solutions.

## 1. Introduction

With the advent and rapid advancement of 5G technology, the increasingly complex electromagnetic environment poses significant challenges to the operation of electronic devices and human safety [1,2,3]. In recent years, electromagnetic microwave-absorbing materials have garnered considerable attention as an effective solution to mitigate electromagnetic wave pollution. These materials are required to be lightweight, thin, possess a wide effective absorption band (EAB), and exhibit excellent absorption loss values. Foam materials, characterized by their low density, help to significantly reduce the structural weight [4,5,6]. Their high porosity facilitates the absorption of electromagnetic waves and minimizes reflection. By adjusting the porosity, pore size, and material composition, the electromagnetic properties of foam materials can be optimized to meet the absorption requirements of different frequency bands. Furthermore, doping with conductive particles, magnetic materials, or other nanomaterials can further enhance the electromagnetic wave-absorption capabilities and other functionalities of foam materials [7,8]. Owing to these advantages, foam materials have garnered considerable attention in the field of electromagnetic wave absorption.

Among various foam electromagnetic absorbing materials, including nickel foam [9,10,11], cement foam [12,13,14], aluminum foam [15,16], polyurethane foam [17,18,19], and carbon-based foam [20,21,22,23,24,25], which have emerged as a focal point in the field of electromagnetic (EM) wave absorption, carbon-based foam materials are extensively utilized in electromagnetic (EM) wave absorption due to their high specific surface area, controllable dielectric properties, and facile element doping (N, S, and P) [26,27,28,29]. Common examples include graphene, multi-walled carbon nanotubes, carbon fibers, and organic or biomass-derived carbon. These materials are characterized by a high loss tangent angle and absorb electromagnetic waves through electron polarization or interface polarization attenuation mechanisms. Glucose, a type of biomass carbon source, offers a greener and simpler carbonization process, forming graphite-like carbon materials with high conductivity, unique structures, and excellent impedance matching. Du et al. [30] reported that C-doped SiC with a heterogeneous structure was synthesized using the solvothermal method. The dielectric properties of this material were controlled by the glucose content, resulting in an exceptional microwave-absorption capacity. Hou et al. [31] coated glucose on the surface of WS_2_ and achieved EM wave-absorption performance adjustments through various carbonization temperatures. At a carbonization temperature of 800 °C, the absorption loss reached −5.52 GHz with an absorption loss of 51.40 dB.

Glucose as a composite material has been widely reported in the field of electromagnetic wave absorption, primarily synthesized through hydrothermal and solvothermal methods as carbon dielectric materials. However, research on the electromagnetic wave-absorbing properties of glucose self-carbonization materials is still relatively scarce. In this study, we proposed a simple transition metal ion-catalyzed glucose-blowing method combined with high-temperature carbonization to produce biomass-derived carbon foams. The introduction of a small amount of metal salts disrupts the local microstructure symmetry, creating additional electric dipoles and enhancing the dipole polarization loss of the material. Additionally, urea acts both as a pore-forming agent and an N-doping agent, further improving the electromagnetic wave-absorption performance of the carbon materials. This approach significantly enhances the absorption bandwidth of the materials, presenting an innovative strategy for the synthesis of electromagnetic wave absorbers with superior microwave-absorption properties.

## 2. Experimental Section and Characterization

### 2.1. Materials

Cobalt chloride hexahydrate (CoCl_2_•6H_2_O, 99%) and iron chloride (FeCl_3_, 99%) were sourced from Shanghai Aladdin Biochemical Technology Co., Ltd. (Shanghai, China). Additionally, glucose monohydrate (C_6_H_12_O_6_•H_2_O, USP) and urea (CH_4_N_2_O, 99%) were procured from Shanghai MackLin Biochemical Technology Co. (Shanghai, China). All chemicals were utilized as received without further purification.

### 2.2. Synthesis of Metal-Containing, Glucose-Derived Carbon Foam (GCF-M)

C_6_H_12_O_6_•H_2_O (25 mmol), urea (17 mmol), CoCl_2_•6H_2_O (0.2 mmol), and FeCl_3_ (0.2 mmol) were weighed and dissolved in 5 mL of deionized water. After ultrasonic dispersion for 30 min, a clear and homogeneous solution was obtained. This solution was then pre-foamed at 140 °C for 8 h to create a metal-containing porous glucose foam (GF-CoFe). Following this, a metal-containing carbon foam derived from glucose was produced using heat treatment at 700 °C for 3 h in an argon atmosphere and designated as GCF-CoFe. Metal-containing, glucose-derived carbon foams containing only Co ions (0.4 mmol) and Fe ions (0.04 mmol) were named GCF-Co and GCF-Fe, respectively. The pure glucose-derived carbon foam without any metal ions is referred to as GCF.

### 2.3. Characterization

The morphology and structure of the samples were examined using Scanning Electron Microscopy (SEM, Carl Zeiss Sigma 500, ZEISS, Oberkochen, Germany) and Transmission Electron Microscopy (TEM, FEI-Tecnai G2 F-20, FEI, Hillsboro, OR, USA). Energy-Dispersive Spectroscopy (EDS, FEI-Tecnai G2 F-20, FEI, Hillsboro, OR, USA) was employed to analyze the elemental composition and determine the composition of the sample surfaces. The phase composition and crystal structure of the carbon foam samples were investigated using X-ray diffraction (XRD, Shimadzu, Kyoto, Japan) with Cu Kα radiation (40 kV, 30 mA, 2° min^−1^, from 5° to 90°). Raman spectroscopy was conducted using a Thermo Scientific DXR 2Xi (Thermo Fisher Scientific, Waltham, MA, USA) equipped with a 532 nm laser to obtain the structural information of the carbon foams and to analyze the effect of metal doping on the carbon material’s structure. The magnetic properties of the metal-containing, glucose-derived carbon foams were examined using a vibrating sample magnetometer (VSM, LakeShore 8604,QUANTUM Scientific Instruments Trading Co., LTD, Beijing, China) under a maximum applied magnetic field of 20 kOe to obtain magnetization hysteresis curves, evaluating the impact of metal doping on the magnetic properties.

### 2.4. EM Parameter Measurements

The materials for Electromagnetic Absorption (EMA) measurements were meticulously prepared by uniformly mixing the sample with paraffin wax at a mass ratio of 20 wt.% sample to 80 wt.% paraffin wax. This homogeneous mixture was then pressed into molds to form coaxial rings. The resulting ring-shaped samples had an outer diameter of 7.00 mm and an inner diameter of 3.04 mm. Electromagnetic testing was conducted using a Keysight N5222B Vector Network Analyzer (Keysight Technologies, Santa Rosa, CA, USA), covering a frequency range from 2 to 18 GHz.

The reflection loss (*RL*) was calculated using the real and imaginary parts of the permeability and dielectric constant based on transmission line theory [32,33]. The equation is expressed as Equations (1) and (2). Here, *Z_in_* is the normalized input impedance, *Z*_0_ is the impedance of free space, *f* is the frequency of EM waves, *d* is the thickness of the material, and *c* represents the velocity of electromagnetic waves in free space [32,33].

(1) $$RL\left(dB\right)=20lg\left|\frac{Z_{in}-1}{Z_{in}+1}\right|$$

(2) $$Z_{in}=\sqrt{\frac{\mu_{r}}{\varepsilon_{r}}}\tanh\left[j\frac{2\pi fd}{c}\sqrt{\mu_{r}\varepsilon_{r}}\right]$$

In order to obtain effective electromagnetic wave absorption, the materials not only need a high attenuation constant but should also have appropriate impedance characteristics. The impedance-matching characteristics (*Z*) of samples can be calculated using the equations below [31], where *Z_in_* represents internal impedance, and *Z*_0_ represents free space impedance. When *Z* equals or approaches 1, the electromagnetic wave will not be reflected on the surface of the absorber, and all incident waves will reflect into the absorber. At the same time, the absolute value of reflection loss will also reach the maximum. If the impedance does not match, the vast majority of incident electromagnetic waves will reflect or directly pass through the absorber without loss, which will lead to *RL* at the bottom of the pole, even if they have excellent dielectric and magnetic losses [34].

(3) $$Z=\left|Z_{in}/Z_{0}\right|$$

The attenuation constant (*α*), a crucial parameter for evaluating the material’s ability to attenuate electromagnetic waves, can be calculated as Equation (4).

(4) $$\alpha =\frac{\sqrt{2}\pi f}{c}\times \sqrt{\left(\mu^{\prime \prime }\varepsilon^{\prime \prime }-\mu^{\prime }\varepsilon^{\prime }\right)+\sqrt{{\left(\mu^{\prime }\varepsilon^{\prime \prime }+\mu^{\prime \prime }\varepsilon^{\prime }\right)}^{2}+{\left(\mu^{\prime \prime }\varepsilon^{\prime \prime }-\mu^{\prime }\varepsilon^{\prime }\right)}^{2}}}$$

## 3. Results and Discussion

**Scheme 1:**
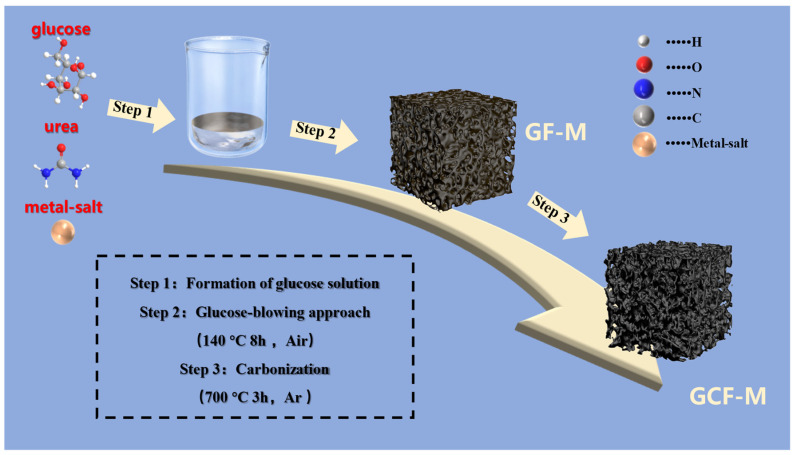
Schematic representation of the synthetic process for GCF-M structures.

The synthesis process of the biomass-derived carbon is schematically illustrated in Scheme 1. Initially, glucose, urea, and metal salts (CoCl_2_•6H_2_O and FeCl_3_) are dissolved in deionized water to create a homogeneous solution. This mixture is then pre-foamed at 140 °C for 8 h. During this process, glucose gradually polymerizes, while urea decomposes slowly, generating gas that causes the molten syrup to expand into large bubbles, thus forming a porous structure. The metal salt serves as a catalyst for the glucose polymerization and becomes uniformly encapsulated by the molten syrup [35,36]. Subsequent carbonization at 700 °C in an argon atmosphere yields a metal-containing, glucose-derived carbon foam.

**Figure 1:**
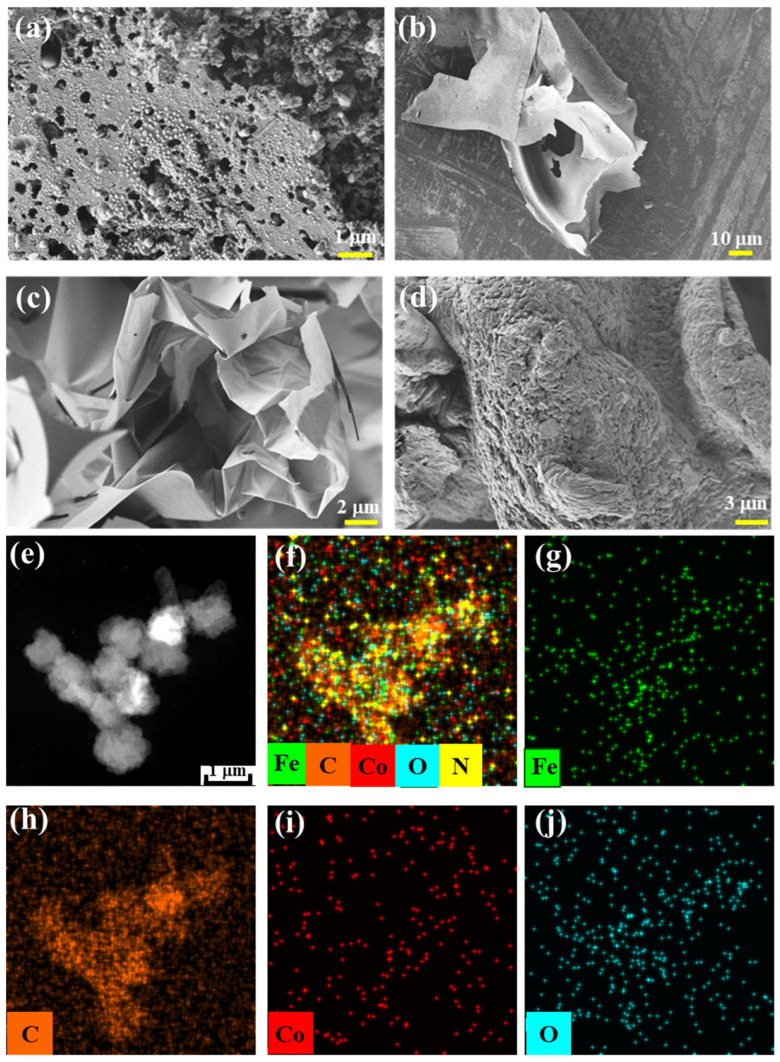
SEM images of GCF-CoFe (**a**), GCF-Co (**b**), GCF-Fe (**c**), and GCF (**d**); HAADF image (**e**) and elemental distribution maps (**f**–**j**) of GCF-CoFe.

Varila et al. [37] reported the catalytic preparation of glucose foam using transition metal ions. The resulting glucose foam volume varied significantly depending on the metal ion used, with GF-Fe exhibiting the greatest volume, followed by GF-CoFe, and then GF-Co. All of these metals contain foams that have a larger volume compared to GF, which was produced without any metal ions. Based on the transition metal-catalyzed synthesis of glucose foam, we further prepared glucose carbon foam (GCF) via high-temperature pyrolysis in an argon atmosphere. The SEM and TEM images of the metal-containing, glucose-derived carbon foams are presented in Figure 1. These images also clearly show that the foam volume significantly increases when catalyzed by Fe/Co metal ions, demonstrating a superior performance compared to the foam produced without catalysts. From the TEM and elemental distribution images of GCF-CoFe (Figure 1e–j), it can be observed that the contents of the elements Co and Fe are relatively low and are evenly distributed on the surface of the material.

**Figure 2:**
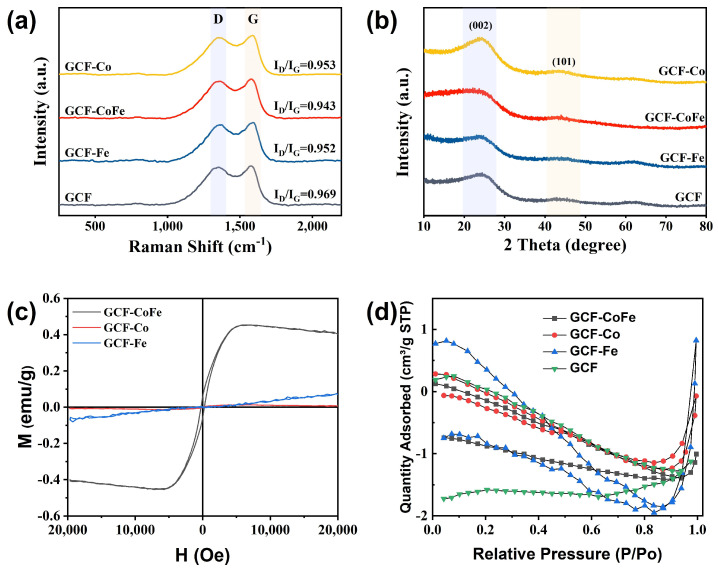
Raman spectra (**a**), X-ray diffraction spectra (**b**), magnetic hysteresis loops (**c**), and N_2_ adsorption–desorption isotherms (**d**) of the samples.

To further analyze the composition and structure of glucose-derived carbon foam, Raman spectroscopy, X-ray diffraction (XRD), magnetic hysteresis loops, and N_2_ adsorption–desorption isotherms were employed (Figure 2). The Raman spectra of all samples are shown in Figure 2a. The typical characteristic peaks of carbon at 1349 cm^−1^ and 1585 cm^−1^, corresponding to the D band and G band, respectively, were observed [38]. The strength ratio of *I*_*D*_/*I*_*G*_ is an indicator of the degree of graphitization in materials. The values for GCF-Co (0.962), GCF-CoFe (0.984), and GCF-Fe (0.966) were higher than that of GCF (0.960), suggesting a greater number of defects in the material structure due to the introduction of metal ions. Specifically, GCF-CoFe exhibited the highest ratio, indicating a significant propensity for defect polarization. The 100–800 cm^−1^ region of the Raman spectrum typically features peaks characteristic of metal particles or complexes [39]. However, no such peaks were observed, likely due to the low metal content present. The XRD pattern of glucose-derived carbon foam revealed two broad diffraction peaks at 24.4° and 43.8°, corresponding to the (002) and (101) crystal planes of graphite carbon, respectively (Figure 2b). This observation confirms the successful transformation of glucose into a graphite-like carbon structure through carbonization. Importantly, no characteristic peaks of metals or metal compounds were detected in the spectra, which can be attributed to the low concentration of metal ions. These findings highlight the structural nuances imparted by metal ion incorporation, leading to an increased number of defects and enhanced defect polarization, particularly in the GCF-CoFe sample. This structural evolution is crucial, as it affects the electromagnetic properties and potential applications of these materials.

Figure 2c shows the hysteresis loops of the GCF-M samples measured at room temperature. The values of specific saturation magnetization (Ms) of GCF-Co, GCF-Fe, and GCF-CoFe were 0.006, 0.065, and 0.44 emu/g, respectively. All samples exhibit a relatively low magnetic performance, which is primarily related to the low content of magnetic metals. The N_2_ adsorption–desorption isotherms of all samples, shown in Figure 2d, indicate that they have relatively small specific surface areas, suggesting the presence of mainly macropores rather than mesopores.

**Figure 3:**
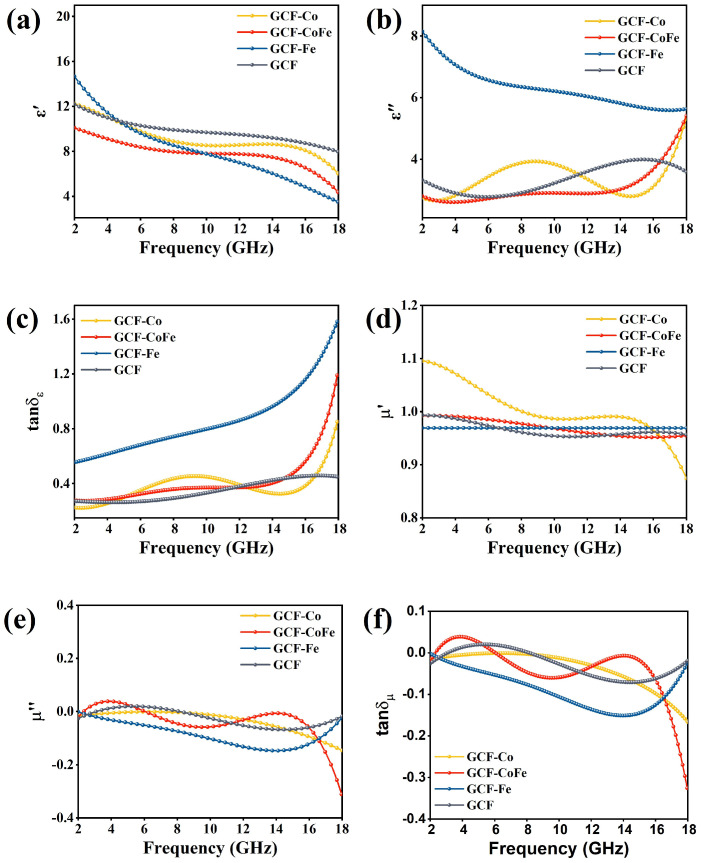
Real (**a**) and imaginary (**b**) parts of permittivity, loss tangent of permittivity (tan*δε*) (**c**), real (**d**) and imaginary (**e**) parts of permeability, and loss tangent of permeability (tan*δμ*) (**f**) of the samples.

When electromagnetic waves interact with the molecular or electronic structure of an absorber, the incident energy is transformed into heat energy within the material. This process leads to energy attenuation and the absorption of electromagnetic waves [40,41]. The relative complex permittivity (*ε*_r_ = *ε*′ − *jε*″) and the relative complex permeability (*μ*_r_ = *μ*′ − *jμ*″) are essential parameters for evaluating the microwave-absorption performances of materials. The real parts of these parameters represent the material’s ability to store energy, while the imaginary parts represent its ability to dissipate electromagnetic energy. Figure 3 illustrate the electromagnetic parameters for all samples within the 2–18 GHz frequency range. The real parts of permittivity (*ε*′) for all samples decrease with an increasing frequency. Conversely, the imaginary parts of permittivity (*ε*″) for GCF-Co, GCF-CoFe, and GCF increase with frequency. However, GCF-Fe exhibits the highest imaginary part value in the low-frequency region, which gradually decreases, approaching the values of GCF-Co and GCF-CoFe at 18 GHz. The imaginary part of the dielectric constant of GCF-Fe is notably large, primarily due to the material’s high conductivity (Equation (5)). This elevated conductivity results in a reduced skin depth of the electromagnetic wave on the material’s surface (see Equation (6)), causing increased reflection of the electromagnetic wave on the surface rather than penetration into the material.

(5) $$\varepsilon^{\prime \prime }\approx \sigma /\varepsilon_{0}\pi f$$

(6) $$\delta =\sqrt{\frac{1}{\pi f\mu \sigma }}$$

The values of *μ*′ and *μ*″ of all samples are close to 1 and 0, respectively, indicating that the magnetic loss can be ignored. Therefore, it can be determined that the absorbing properties of all samples mainly depend on the relative complex permittivity [42,43].

The tan*δε* values for GCF-Co range from 0.22 to 0.85, for GCF-CoFe from 0.27 to 1.19, for GCF-Fe from 0.5 to 1.6, and for GCF from 0.27 to 0.45. The dielectric loss tangent (tan*δε*) of GCF-Fe is stronger than that of GCF-CoFe, which is stronger than that of GCF-Co, which, in turn, is stronger than that of GCF. The tan*δμ* values for GCF-Co range from −0.01 to −0.17, for GCF-CoFe from −0.01 to −0.32, for GCF-Fe from −0.01 to −0.03, and for GCF from −0.02 to −0.02. The magnetic loss tangents (tan*δμ*) of all the materials are relatively small, while the dielectric loss tangents are significantly higher than the magnetic loss tangents. This indicates that the primary wave-absorption mechanism for all glucose carbon foam materials is dielectric loss.

**Figure 4:**
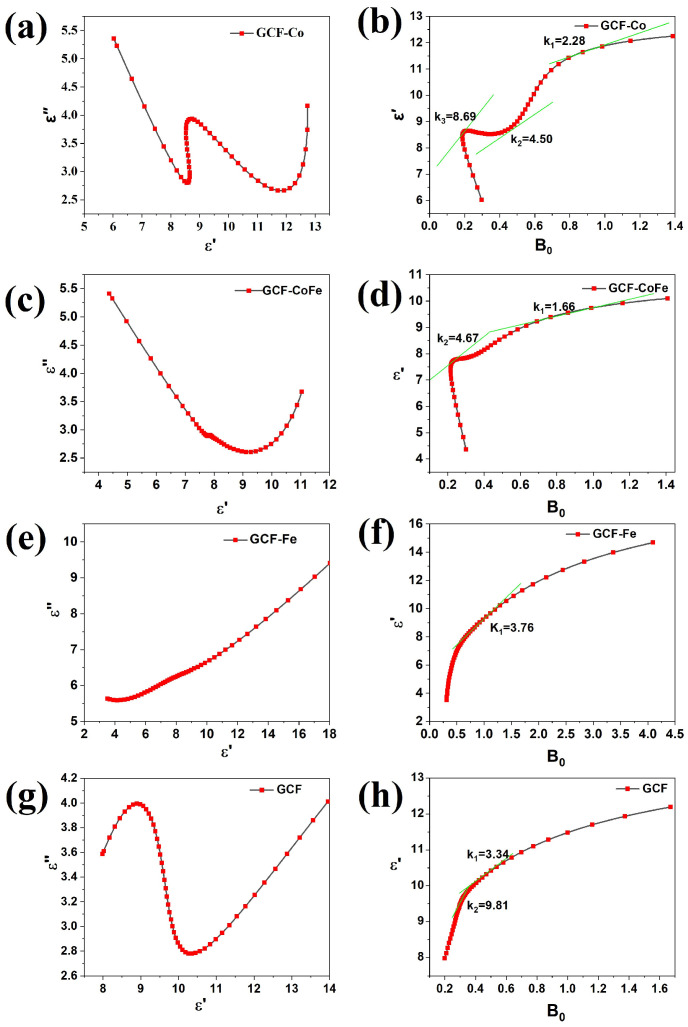
The Cole–Cole plots (**a**,**c**,**e**,**g**) and ε′-B_0_ plots (**b**,**d**,**f**,**h**) of the samples.

The Cole–Cole plot (see Figure 4) can be used to study the relaxation process of the sample. The relationship between *ε*′and *ε*″ is given by Equation (8).

(7) $${\left(\varepsilon^{\prime }-\frac{\varepsilon_{\infty }+\varepsilon_{s}}{2}\right)}^{2}+{\left(\varepsilon^{\prime \prime }\right)}^{2}={\left(\frac{\varepsilon_{s}-\varepsilon_{\infty }}{2}\right)}^{2}$$

(8) $$\varepsilon^{\prime }=\frac{\varepsilon^{\prime \prime }}{2\pi f\tau }+\varepsilon_{\infty }$$

Assuming $B_{0}=\varepsilon^{\prime \prime }/f$, the slope of Equation (8) can be expressed as k=1/2πτ. The Debye relaxation frequency $f_{r}=1/2\pi \tau$ is equal to the slope in Equation (8). The Cole–Cole plot indicates the presence of multiple relaxation processes in the carbon foam. The GCF-Co sample exhibits three Debye circles, with Debye polarization frequencies of 2.28 GHz, 4.50 GHz, and 8.69 GHz, respectively. The GCF-CoFe sample shows two Debye circles, with Debye polarization frequencies of 1.66 GHz and 4.67 GHz. The GCF-Fe sample has one Debye circle, with a Debye polarization frequency of 3.76 GHz. Lastly, the GCF sample exhibits two Debye circles, with Debye polarization frequencies of 3.34 GHz and 9.81 GHz. The incorporation of metal ions affects the relaxation process of glucose-derived carbon foams. The observed Debye circles indicate that different relaxation processes occur in each sample, attributed to the interactions between metal ions and the carbon material with the electromagnetic field.

**Figure 5:**
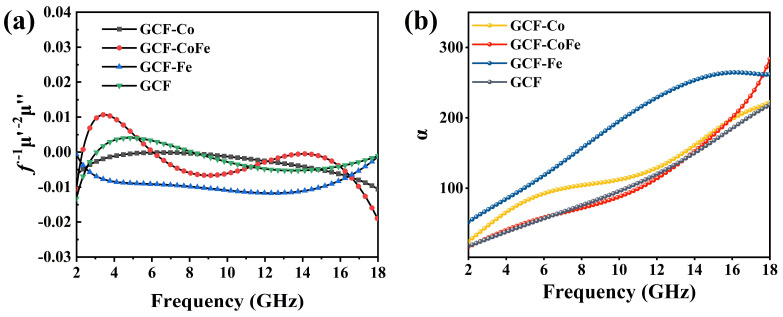
(**a**) The $f^{-1}{\left(\mu^{\prime }\right)}^{-2}\mu^{\prime \prime }$ and (**b**) attenuation constant of the samples.

The $C_{0}$ curves of the four glucose-derived carbon foams, as depicted in Figure 5a, reveal important insights into their magnetic loss mechanisms. According to the relationship $C_{0}=f^{-1}{\left(\mu^{\prime }\right)}^{-2}\mu^{\prime \prime }=2\pi \mu_{0}d^{2}\sigma$, if the value remains constant with varying frequency, it signifies that the magnetic loss is predominantly due to eddy current loss. For GCF-Fe, $C_{0}$ remains nearly constant over the 4–14 GHz range, indicating that eddy current loss is the dominant mechanism. Conversely, for the other glucose-derived carbon foams, magnetic loss is primarily attributed to natural resonance and exchange resonance.

**Figure 6:**
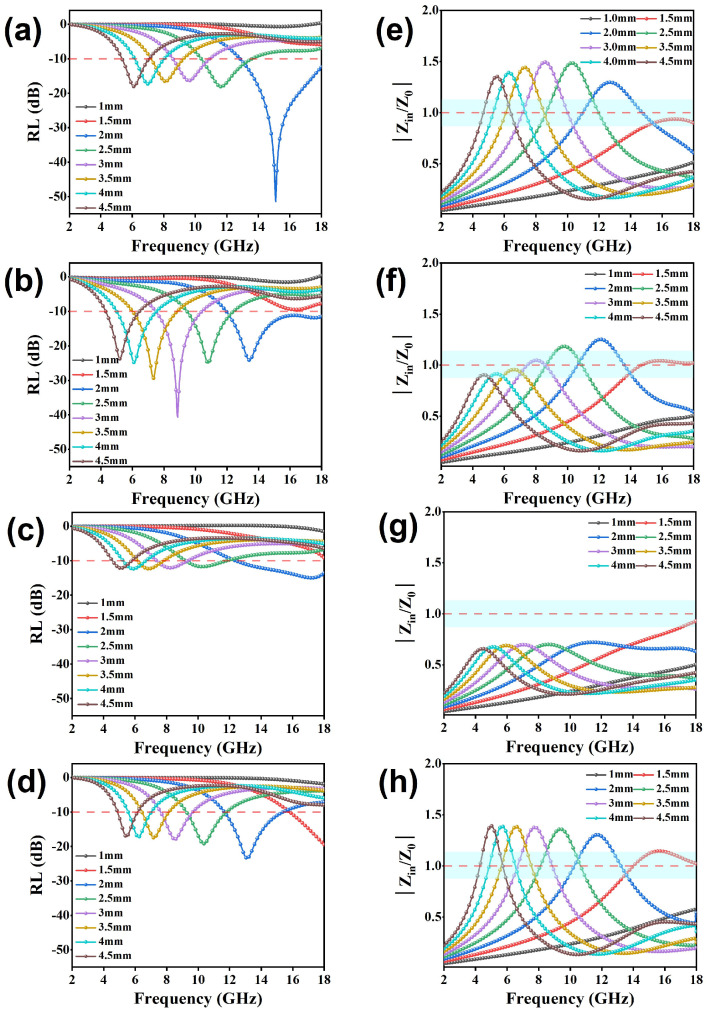
Reflection loss values, effective bandwidth, and modulus curves of *Z_in_*/*Z*_0_ for GCF-CoFe (**a**,**e**), GCF-Co (**b**,**f**), GCF-Fe (**c**,**g**), and GCF (**d**,**h**).

In order to further explore the dielectric loss ability of the material, attenuation constant (*α*) curves were observed. It was found that all samples exhibited an upward trend, indicating inherent absorption properties in all samples [44,45]. This upward trend suggests that the materials have effective interactions with electromagnetic waves, leading to energy dissipation. Further analysis revealed variations in the attenuation constants among different samples, which could be attributed to the specific interactions between the incorporated metal ions and the carbon matrix [46,47]. In general, a high attenuation constant correlates with a superior attenuation capacity [48,49]. As shown in Figure 5b, the attenuation constants (*α*) of glucose-derived carbon foams are ranked as follows: *α*(GCF-Fe) > *α*(GCF-Co) > *α*(GCF-CoFe) ≈ *α*(GCF). These results indicate that GCF-Fe exhibits the highest attenuation constant, suggesting it has the most effective electromagnetic wave-absorption and -dissipation properties. This order highlights the metal ions’ significant influence on the dielectric loss performance of glucose-derived carbon foams. Although GCF-Fe exhibits the highest Electromagnetic Absorption coefficient and its dielectric loss tangent is significantly greater than that of the other three materials, its impedance matching (see Figure 6g) is not close to 1. This indicates that the excessively large dielectric loss tangent of GCF-Fe causes electromagnetic waves to be predominantly reflected on the surface of the material rather than penetrating into its interior. Consequently, while GCF-Fe has superior absorption properties, the poor impedance matching hinders its overall wave-absorbing efficiency by preventing effective energy dissipation within the material [50].

**Figure 7:**
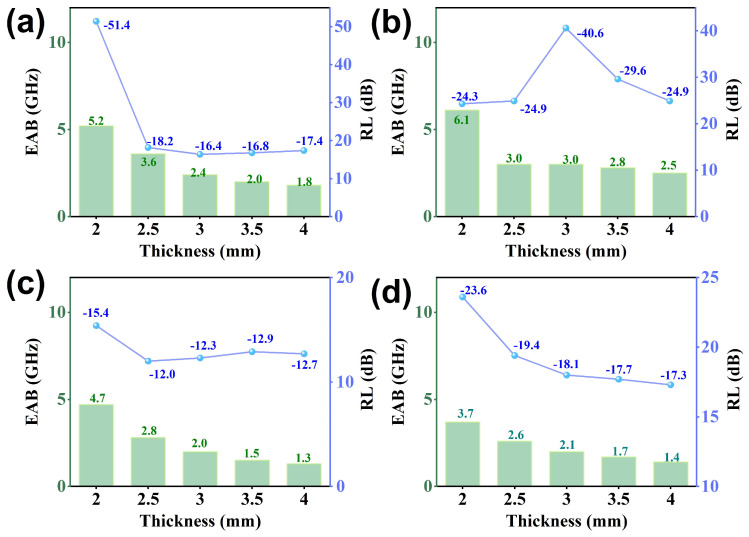
Effective bandwidth for GCF-CoFe (**a**), GCF-Co (**b**), GCF-Fe (**c**), and GCF (**d**).

Figure 6a–d present the reflection loss (*RL*) curves for different thicknesses of all samples. The *RL* values for all samples fall below −10 dB, indicating an absorption capability of at least 90% for incident electromagnetic (EM) waves. Notably, the GCF-CoFe sample exhibits a superior EM wave-absorption performance, achieving a minimum *RL* value of −51.4 dB at 15.4 GHz with a thickness of 2 mm. In comparison, the optimum *RL* values for GCF and GCF-Co are −23.6 dB at 12.8 GHz and −40.6 dB at 8.9 GHz, respectively. On the other hand, GCF-Fe demonstrates the least effective microwave absorption, with an *RL* of −15.4 dB at 17.2 GHz, despite possessing the highest attenuation constant among the samples. Further analysis of the impedance-matching curves (Figure 6e–h) reveals that GCF-Fe displays the best impedance-matching characteristics, which is intriguing given its lower dielectric constant compared to GCF-CoFe. This suggests that impedance matching plays a crucial role in determining the overall absorption efficiency. Moreover, the effective absorption bandwidth (EAB) for all materials is detailed in Figure 7. The EAB values are 3.7 GHz for GCF, 6.1 GHz for GCF-Co, 5.2 GHz for GCF-CoFe, and 4.7 GHz for GCF-Fe. These results underscore the potential of these materials for applications requiring broad bandwidth absorption of EM waves, with GCF-CoFe and GCF-Co standing out due to their remarkable performance characteristics (which shown in Table 1).

**Figure 8:**
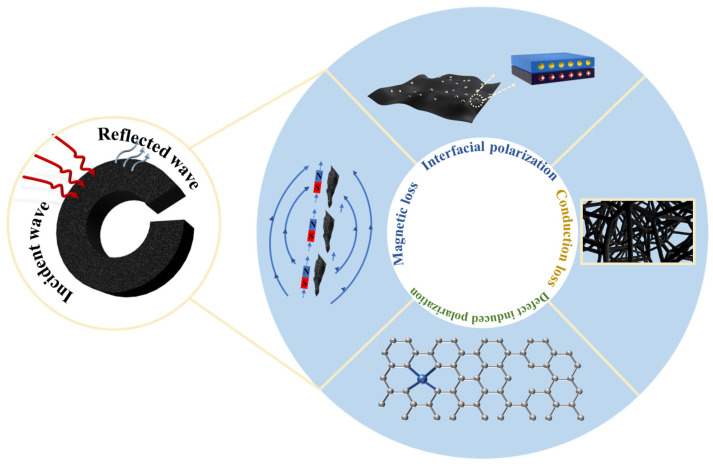
Schematic diagram of the absorption mechanism.

Figure 8 illustrates the absorption mechanism of metal-containing porous carbon foams. When materials exhibit porous, folded, or hollow structural features, they form numerous interfaces with air. The presence of these interfaces results in significant differences in dielectric properties between the material and air, leading to multiple reflections and transmissions of electromagnetic waves as they cross these interfaces, thereby promoting energy dissipation of the electromagnetic waves. Particularly in highly conductive porous materials, their unique porous structure not only enhances impedance matching between the material and electromagnetic waves but also optimizes the propagation efficiency of electromagnetic waves within the material, effectively attenuating the waves. For glucose-derived carbon foam materials, the primary factors influencing electromagnetic energy loss mechanisms include conductive loss, interfacial polarization, dipole polarization, and magnetic loss. These porous carbon materials, with their unique structural characteristics, exhibit tremendous potential in the field of electromagnetic wave absorption, and are of significant practical importance for the development of efficient electromagnetic wave absorption and electromagnetic shielding materials.

## 4. Conclusions 

In this paper, we successfully utilized Fe and Co transition metal ions in a sugar-blowing method to foam natural glucose. This process was followed by high-temperature pyrolysis to prepare metal-containing glucose carbon foam. The optimum *RL* values for GCF, GCF-Co, and GCF-Fe are −23.6 dB at 12.8 GHz, −40.6 dB at 8.9 GHz, and −15.4 dB at 17.2 GHz, respectively. The resulting glucose foam, catalyzed by FeCo bimetallic ions, exhibited exceptional wave-absorption properties due to its porous structure. At a thickness of 2.0 mm, the GCF-CoFe composite achieved an impressive minimum reflection loss (*RL*_min_) of −51.4 dB at 15.11 GHz. Additionally, it demonstrated an effective absorption bandwidth (EAB) spanning 5.20 GHz, ranging from 12.80 GHz to 11.48 GHz. These remarkable properties suggest that this method provides a promising direction for the development of low-density carbon foam wave-absorbing materials. The incorporation of Fe and Co transition metals in the foaming process not only enhances the structural properties of carbon foam but also significantly boosts its electromagnetic wave-absorption capabilities. This innovative approach paves the way for the development of advanced materials with potential applications in electromagnetic interference (EMI) shielding, stealth technology, and other fields requiring efficient wave absorption. Future research will focus on the co-carbonization of glucose with other materials to address the inherent hydrophilicity of glucose-derived carbon materials. This strategy aims to improve the adaptability of these materials in complex environments, thereby broadening their applicability and effectiveness.

## Figures and Tables

**Table 1:** Comparison of absorbing performance parameters for the samples.

Samples	Thickness mm	*RL*_min_/dB (Corresponding Frequency GHz)	Frequency Range/GHz	EAB/GHz	Ref.
Co–Ni/C composite	3.3	−34.3 (~9.6)	8.00–12.24	4.24	[46]
Fe–Ni/C composite	5.7	−42.3 (17.1)	15.20–18.00	2.80	[46]
Fe-N-CNS	1.0	−37.33 (11.41)	9.44–16.17	6.73	[47]
Graphene foam	10	−34.0 (13.1)	~3.30–18.00	14.7	[48]
Graphene Aerogel	1.14	−50.3 (24.29)	21.23–26.50	5.27	[49]
Fe_3_O_4_@C/GA	0.99	−54.0 (19.87)	18.00–24.50	6.50	[49]
MMT/Fe_3_O_4_/PPy	3.5	−41.35 (11.52)	6.47–15.39	8.92	[50]
GCF-Co	2.0	−24.3 (13.40)	12.80–18.00	6.10	This work
GCF-CoFe	2.0	−51.4 (15.11)	12.80–18.00	5.20	This work
GCF-Fe	2.0	−15.4 (17.14)	13.3–18.00	4.7	This work

## Data Availability

The original contributions presented in the study are included in the article, further inquiries can be directed to the corresponding authors.
